# Diversity and Plant Growth Promotion of Fungal Endophytes in Five Halophytes from the Buan Salt Marsh

**DOI:** 10.4014/jmb.2012.12041

**Published:** 2021-01-04

**Authors:** Irina Khalmuratova, Doo-Ho Choi, Hyeok-Jun Yoon, Tae-Myung Yoon, Jong-Guk Kim

**Affiliations:** 1 School of Life Science and Biotechnology, Kyungpook National University, Daegu 701-701, Republic of Korea; 2 Department of Horticultural Science, Kyungpook National University, Daegu 41566, Republic of Korea

**Keywords:** Coastal salt marsh plants, fungal endophytes, Buan salt marsh, growth promotion, gibberellin

## Abstract

The diversity and plant growth-promoting ability of fungal endophytes that are associated with five halophytic plant species (*Phragmites australis*, *Suaeda australis*, *Limonium tetragonum*, *Suaeda glauca* Bunge, and *Suaeda maritima*) growing in the Buan salt marsh on the west coast of South Korea have been explored. About 188 fungal strains were isolated from these plant samples’ roots and were then studied with the use of the internal transcribed spacer (ITS) region (ITS1-5.8S-ITS2). The endophytic fungal strains belonged to 33 genera. *Alternaria* (18%) and *Fusarium* (12.8%), of the classes Dothideomycetes and Sordariomycetes, were most rampant in the coastal salt marsh plants. There was a higher diversity in fungal endophytes that are isolated from *S. glauca* Bunge than in isolates from other coastal salt marsh plants. Plant growth-promoting experiments with the use of Waito-C rice seedlings show that some of the fungal strains could encourage a more efficient growth than others. Furthermore, gibberellins (GAs) GA_1_, GA_3_, and GA_9_ were seen in the Sa-1-4-3 isolate (*Acrostalagmus luteoalbus*) culture filtrate with a gas chromatography/mass spectrometry.

## Introduction

The world’s population is estimated to rise to 9 billion by 2050 [1]. There is a need to increase food production under less than optimal conditions due to population growth and climate change. In the mid- to late twentieth century, the cultivation efforts’ purpose is to improve crop varieties, introduce hybrids, and increase agricultural productions in terms of fertilizer, crop management practices, herbicides, water delivery systems, and pesticides resulted in the “Green Revolution” [2, 3]. Worldwide, there is a similar global challenge, leading to a need of breed-improved varieties as well as agricultural production practices [4]. The expansion of agricultural production to marginal lands and the effects of global climate change also need an increased biotic and abiotic stress tolerance and efficient nutrient utilization in crop plants for the future global food requirements to be met.

Endophytic fungi are organisms that are found anywhere (intercellularly or intracellularly in plants from nearly all genera of Kingdom Plantae). These fungi live in host plants for at least a portion of their lives without generating any immediate overt disease symptoms. These associations can encourage tissue differentiation and plant growth and can help in managing abiotic and biotic stresses to which the host plants are subjected [5, 6]. In addition, endophytic fungi may prevent pathogenic organisms and provide nutrients, benefiting the plant host [7]. These well-distributed fungi form diverse plant associations and thus constitute outstanding sources of new bioactive secondary metabolites [8]. Accordingly, there are several new bioactive compounds with insecticidal, antimicrobial, cytotoxic, and anticancer activities that have been separated from endophytic fungi recently [9, 10].

Plant signaling compounds, also called phytohormones, regulate plant responses to environmental change as well as control plant growth and development [11]. Notably, recent studies have reported that certain endophytes encourage host plant growth through the synthesis of phytohormones, for example, gibberellins (GAs), cytokinins, and indole-3-acetic acid (IAA) [12-14]. Indeed, endophytic fungi promote plant growth by secreting gibberellins in the rhizosphere of their hosts, which leads to an increase in plant biomass production as well as disease resistance. Apart from this, some endophytic fungi secrete both IAA and GAs into culture media [15].

This research surveys both distribution and diversity of fungal endophytes in a certain coastal region of Korea. We have investigated fungal isolates’ capacity to encourage growth of Waito-C rice seedlings and have identified whether secondary metabolites like gibberellins were seen in fungal culture filtrates.

## Materials and Methods

### Plant Materials and Sampling Sites

Healthy plants and roots of *Phragmites australis*, *Suaeda australis*, *Limonium tetragonum*, *Suaeda glauca* Bunge, and *Suaeda maritima* were gathered from different places in the Buan salt marsh in South Korea. The samples were carefully sealed in sterile plastic bags and were then processed in the laboratory within 24 hours of collection. The scientific names, codes, and local sites of the five plant species are listed in Table 1.

### Sterilization and Isolation of Endophytes from the Roots of Halophytes

Fungal endophytes were isolated from healthy roots of halophytic plants that were gathered from the salt marsh. Each plant’s root samples were washed with tap water, cut into 2–2.5-cm-long segments, and treated with Tween 80 solution for 10 min on a shaker with 160 rotations per minute (rpm). Afterward, root segments were incubated in a solution of 1% (w/v) perchloric acid for 10 min and then rinsed with double-distilled water [16, 17]. Then, they were dehydrated for 5–6 min at a temperature of 22°C on a clean bench, and two to three root segments were placed in a 90-mm Petri plate with Hagem minimal media that contain 80 ppm streptomycin. Samples were incubated at 25°C in dark conditions until there is growth of fungi from the root segments seen [18, 19]. Lastly, pure fungal strains were separated from the root segments and were kept on potato dextrose agar at 25°C [20].

### DNA Extraction, PCR Amplification, and the Identification of Fungal Strains

Fungal endophyte cultures were grown in an Erlenmeyer flask that contains 50 ml potato dextrose broth for 7–10 days at a temperature of 26°C on a shaker at 120 rpm. All the 188 lyophilized endophyte samples were known. There was a fungal genomic DNA extraction using a DNeasy Plant Mini Kit (Qiagen, USA). There was identification of fungi performed by sequencing the internal transcribed spacer (ITS) region with the universal primers ITS-1 (5′-TCCGTAGGTGAACCTGCGG-3′) and ITS-4 (5′-TCCTCCGCTTATTGATATGC-3′). Reaction cycling comprised of an initial denaturation step at 95°C for 2 min, which is then followed by 35 cycles of denaturation at 95°C for 30 sec, annealing at 55°C for 1 min, and extension at 72°C for 1 min. The final extension was done at 72°C for 7 min. PCR products were electrophoresed on agarose gels with an ethidium bromide stain and purified using the QIAquick PCR purification kit (Qiagen). The products were then sequenced with the use of the ABI PRISM BigDye terminator cycle sequencing kit (PE Biosystems, USA) on an ABI 310 DNA automated sequencer (Perkin, USA). The sequences were identified with the use of the BLAST (Basic Local Alignment Search Tool) tool of the National Center for Biotechnology Information (NCBI).

### Statistical Analyses

The fungal endophytes’ diversity at the genus level was indicated by the Shannon diversity index (H’), Fisher’s alpha index (α), and Simpson’s index of diversity. Richness was assessed by Menhinick’s richness index (Dmn) and Margalef ’s index (Dmg) [21, 22]. The Menhinick’s index was calculated through the following formula: $\mathrm{Dmm}=S/\sqrt{N}$; Dmg=(S−1)/ln(N), where S is the number of genera in a sample, and N is the total number of individuals in a community. Both indices ranged from 0 to ∞.

The genus diversity was evaluated using the Shannon diversity index (*H*’), Fisher’s alpha index (α), and Simpson’s index of diversity [23]. Fisher’s alpha index (α) was calculated as follow; S=α·ln(1+N/α), where S is the number of genera, and N is the total number of individuals. The formula for Shannon’s diversity index is $H'=-\Sigma_{i=1}^{R}\mathrm{pi}\cdot \mathrm{lnpi}$, where *pi* is the proportion of individuals found in genera *i* in a sample. The values of the Shannon diversity index generally range from 1.5 to 3.5. Simpson’s index of diversity (*1-D*) was calculated as follow; $D=\Sigma_{i=1}^{R}\mathrm{ni}(\mathrm{ni}-1)/N(N-1)$, where *N* is the total number of individuals in a sample, and *ni* is the number of individuals found in genera *i* in a sample. The magnitude of this index ranges from 0 to 1; the greater the magnitude, the greater the sample diversity.

### Screening of Fungal Cultures on Waito-C rice Seedlings

To test whether the fungi have growth-promoting capacities, Waito-C rice sprouts were exposed to fungal culture filtrates. The fungal strains were grown in the Czapek Dox broth medium on a shaking incubator for 7 days at 25°C and 180 rpm and were harvested using filtration. The harvested fungal culture filtrates were immediately stored at a temperature of −70°C and then lyophilized. The lyophilized culture filtrates were then mixed together with 1 ml of distilled water. Waito-C rice grains were treated with uniconazole for 24 h to lessen gibberellins’ activity in the seed coat. The treated rice seeds were washed and soaked in distilled water until radical emergence occurs, and then the young seedlings were placed in glass tubes with 0.6% water agar medium to grow in a growth chamber [24]. Concentrated, lyophilized culture filtrates (10 microliters) from each fungal isolate were applied to apical meristems after the rice seedlings reached the two-leaf stage. Both the plant and shoot lengths of rice were seen after 1 week application and compared against rice seedlings treated with wild-type *Gibberella fujikuroi*, which was the positive control in this study.

### Analysis of Gibberellins

Gibberellins were extracted from culture filtrates after the incubation of fungal isolates for 7 days in Czapek Dox broth medium that contains 1% (w/v) glucose and peptone. Reverse-phase C_18_ high-performance liquid chromatography followed by gas chromatography/mass spectrometry (GC/MS) with selected ion monitoring (SIM) investigated extracted gibberellins. Three major ions of the supplemented [^2^H_2_] gibberellin internal standards as well gibberellins were simultaneously monitored. The GC/MS data were eventually collected and investigated. The retention time was known with the use of hydrocarbon standards in calculating the Kovats retention index (KRI) value, and peak area ratios between non-deuterated and deuterated gibberellins were used for the quantification of the gibberellins [25].

## Results

### Identification of Endophytic Fungi

There is a total of 188 endophytic fungal strains separated from the roots of five species of plants collected from the Buan salt marsh on the west coast of South Korea. Fifty-two fungal strains from *P. australis*, 40 strains from *S. australis*, 25 strains from *L. tetragonum*, 41 strains from *S. glauca* Bunge, and 30 strains from *S. maritima* were isolated.

The endophytic fungi nucleotide sequences from each plant sample were deposited in the NCBI GenBank database under the accession numbers KP018214–KP018265 from *P. australis*, KP018266–KP018305 from *S. australis*, KP018306–KP018330 from *L. tetragonum*, KP018331–KP018371 from *S. glauca* Bunge, and KP018372–KP018401 from *S. maritima* (Table 2).

There was a total of 188 strains categorized into the phyla Ascomycota (186 strains) and Basidiomycota (two strains). The class Dothideomycetes (93 strains) has the largest number of strains, followed by the following classes: Sordariomycetes (56 strains), Eurotiomycetes (36 strains), Exobasidiomycetes (two strains), and Leotiomycetes (one strain). At the genus level, *Alternaria* has the largest proportion of isolates (34 strains) followed by *Fusarium* (24 strains).

**Fig. 1:**
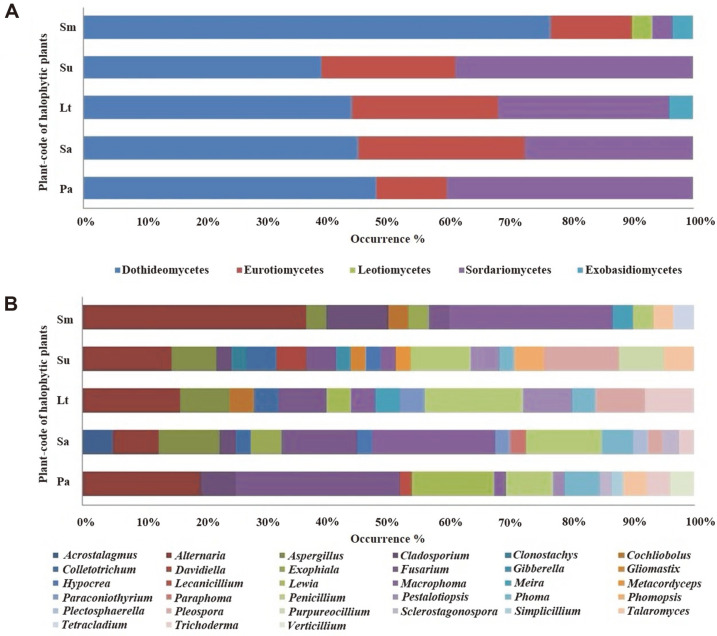
Distribution of fungal isolates in different plant samples at the class (A) and genus (B) levels. Pa, *Phragmites australis*; Sa, *Suaeda australis*; Lt, *Limonium tetragonum*; Su, *Suaeda glauca* Bunge; and Sm, *Suaeda maritima*.

The genus of each strain was observed, and each group’s proportion at the class and genus levels was assessed (Fig. 1). Dothideomycetes has the highest percentage of isolates at the class level. With the exception of isolates from *S. glauca* Bunge, Dothideomycetes is the majority of fungi in every plant sample. At the genus level, *Alternaria* was the most prevalent (18%) fungal isolate, which is followed by *Fusarium* (12.7%).

### Diversity of Endophytic Fungi isolates

About 188 culturable fungal strains were isolated from the roots of five halophytes based on colony morphologies. All endophytic fungi from halophytes belonged to 33 genera in accordance with molecular identification. Fungal isolates were then classified into 14 genera, 16 species, and 19 unclassified strains from *P. australis*; 17 genera, 17 species, and 17 unclassified strains from *S. australis*; 14 genera, 13 species, and 11 unclassified strains from *L. tetragonum*; 19 genera, 14 species, and 17 unclassified strains from *S. glauca* Bunge; and 11 genera, 7 species, and 22 unclassified strains from *S. maritima* (Table 3).

As regards generic diversity, *S. glauca* Bunge had the highest score in Shannon’s index (2.76), Fisher’s (α) (13.75), and Simpson’s index of diversity (0.970). *S. maritima* had the lowest scores in Shannon’s index and Fisher’s (α) (1.86 and 6.26, respectively), and *P. australis* had the lowest Simpson’s index of diversity (0.904). As regards generic richness, *S. glauca* Bunge displayed the highest scores in Menhinick’s index (2.97) and Margalef ’s index (4.85). The lowest scores in Menhinick’s and Margalef ’s indices were in *P. australis* (1.94) and *S. maritima* (2.94), respectively (Table 4).

### Screening for Fungal Metabolites Promoting Plant Growth in Waito-C rice Seedlings

**Fig. 2:**
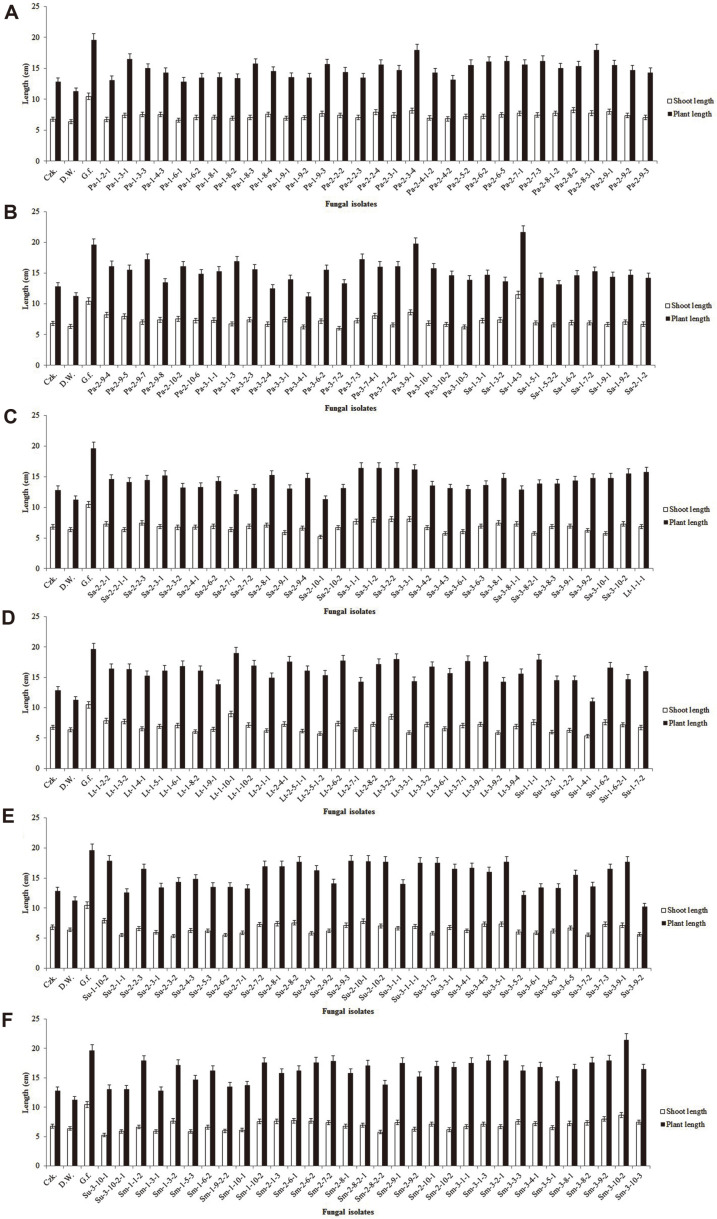
Screening for plant growth promoting of Waito-C rice seedlings with culture filtrates of fungal endophytes isolated from plant samples A-F. Ten microliters of lyophilized culture filtrates was treated to Waito-C rice seedlings. The shoot length and plant length of the Waito-C rice seedlings were measured after 7 days of treatment. The standard deviation from means was calculated using Microsoft Excel.

The fungal culture filtrates were applied on Waito-C rice seedlings, and the seedling lengths were measured after 1 week of fungal culture filtrate application. Out of 188 isolated fungi, 4 fungal isolates considerably encouraged shoot lengths of rice seedlings. The fungal isolate Sa-1-4-3 promoted maximum plant and shoot lengths of 21.6 cm and 11.5 cm, respectively, while culture filtrates Pa-3-9-1, Lt-1-10-1, and Sm-3-10-2 produced plant and shoot lengths of 19.7 and 8.6 cm, 19 and 9 cm, and 21.4 and 8.6 cm, respectively. There was a much lower plant and shoot growth promotion by *Gibberella fujikuroi* than by Sa-1-4-3 (Fig. 2); consequently, Sa-1-4-3 was chosen for further analysis.

### Extraction and Quantification of Gibberellins

**Fig. 3:**
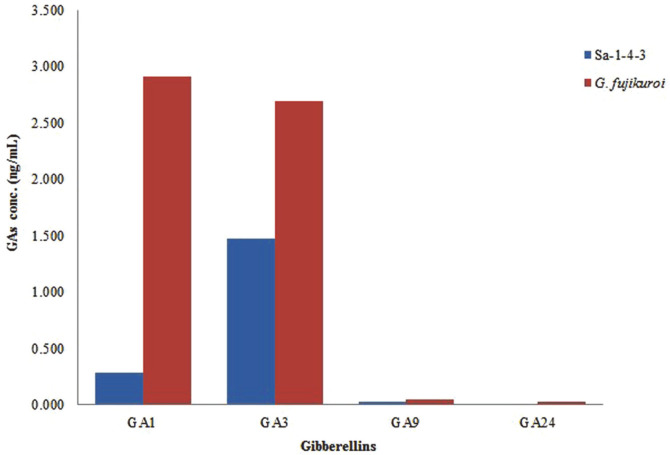
Gibberellins content of fungal culture filtrates of the Sa-1-4-3 strain and wild type *Gibberella fujikuroi*. GC/MS-SIM analysis of culture filtrate extracts from the Sa-1-4-3 fungal isolate detected two bioactive GAs. Sa-1- 4-3 showed the presence of bioactivity of GA1, GA3, and other inactive GA.

After the growth of Sa-1-4-3 fungal strain for 7 days in Czapek Dox broth medium (at 25°C; 180 rpm), the culture was then filtered with the use of a filter paper to obtain a clear supernatant (150 ml). The supernatant was extracted and chromatographed for the detection of gibberellins. The GC/MS-SIM analysis has discovered that different physiologically active and inactive gibberellins have different quantities. The physiologically bioactive gibberellins were GA_1_ (0.285 ng/ml) and GA_3_ (1.479 ng/ml), while the physiologically inactive gibberellins were GA_9_ (0.029 ng/ml) and GA24. GA_3_ was considered to be more abundant than other gibberellins (Fig. 3).

## Discussion

Endophytes reside inside healthy plant tissues, providing shelter and nutrition. In return, these endophytes act as an excellent source of bioactive compounds and functional metabolites that impact both plant health and growth [26-28]. Some of these metabolites prompt resistance mechanisms that protect the plants against different biotic and abiotic stresses; therefore, plant fitness and productivity are increased [29].

In our study, 188 endophytic fungi were isolated from 5 plants growing in the Buan salt marsh and were identified by ITS1, 5.8S, and ITS2 sequencing. Thirty-three genera were recognized among the isolated fungal samples. *Alternaria* and *Fusarium* were the most commonly found fungi. The majority of endophytic fungi belong to the phylum Ascomycota [30, 31]. Recent studies have reported that the genus *Alternaria* was the most prominent fungus found in root, stem, and leaf tissues of *Gossypium hirsutum* [32]. Additionally, both *Alternaria* and *Fusarium* are the most frequently encountered endophytes in different types of plants [32].

Fungal endophytes are well known for their production of a wide range of secondary metabolites and enhancement of plant resistance to environmental stress. The plant growth-enhancing effects of these fungal culture filtrates were confirmed with the use of Waito-C rice seedling bioassays [33, 34]. Although other plants are also valuable for analyze the plant growth-enhancing effects, Waito-C rice has more benefits. Because of small size of plant and lack of gibberellin, uniconazole treated Waito-C rice reacts sensitively to gibberellin from foreign substances and shows rapidly the difference in growth following injection. The use of rice mutant Waito-C, which reduces gibberellin biosynthesis, was very efficient. The Sa-1-4-3 fungal strain has promoted a better plant growth in the Waito-C rice seedlings. These results are the same as those of a previous study wherein *Talaromyces pinophilus* Su-3-4-3, which is isolated from the roots of *S. glauca* Bunge, encouraged the growth in various rice plants [35].

The host plant benefits from the hormones produced by endophytic fungi. Several studies have shown plant growth-promoting characteristics and secretion of secondary metabolites, such as gibberellins, of endophytic fungi, most of which have a relationship with the roots [34, 36]. In growth of plant, many other growth promoting compounds including cytokinins and indole-3-acetic acid are also existing. But since gibberellin is the most representative compound, it was selected as a target material for growth promotion in this study. We have acquired a strain that was initially identified as *Acrostalagmus luteoalbus* (Sa-1-4-3) and detected gibberellin in this culture’s filtrate. According to the result of the Waito-C rice cultivation, the *A. luteoalbus* strain (Sa-1-4-3) showed the better growth contribution of plant length than the *G. fujikuroi*. Based on the period of plant cultivation in this study, it is expected that *A. luteoalbus* will promote early growth of Waito-C rice in particular. The GC/MS-SIM method, an established technique to identify secondary metabolites, analyzed the gibberellins in the culture filtrate of Sa-1-4-3.

In a nutshell, a total of 188 fungi were isolated from roots of five plants that are located in the Buan salt marsh. These fungi were classified into 2 phyla, 5 classes, 10 orders, 19 families, and 33 genera. *Alternaria* and *Fusarium* accounted for more than 30% of all isolates. Endophytic fungi that are isolated from *S. glauca* Bunge are the most diverse. This study informs us on the capacity of *A. luteoalbus* (Sa-1-4-3) to produce gibberellins. These results are expected to promote plant growth in areas with high salt concentrations, which are considered to be of great benefit to crop production and agriculture.

## Figures and Tables

**Table 1:** Geographic coordinates and scientific names of plants native to the Buan salt marsh.

No	Scientific name	Code	Site of collection	Habitat
1	*Phragmites australis*	Pa	35°35'34.69"N / 126°36'2.42"E	Halophytic
2	*Suaeda australis*	Sa	35°35'10.99"N / 126°32'44.70"E	Halophytic
3	*Limonium tetragonum*	Lt	35°35'10.73"N / 126°32'51.40"E	Halophytic
4	*Suaeda glauca* Bunge	Su	35°35'10.80"N / 126°32'51.90"E	Halophytic
5	*Suaeda maritima*	Sm	35°35'34.92"N / 126°36'3.95"E	Halophytic

**Table 2:** Identification of endophytic fungal isolates from roots of plants.

Sample No.	Closely related fungal sequences	Similarity (%)	Accession No.
Pa-1-2-1	*Pestalotiopsis* sp. 1 MJ-2014 (KJ572189)	100	KP018214
Pa-1-3-1	*Alternaria* sp. BOP212b (KC771455)	100	KP018215
Pa-1-3-3	*Fusarium incarnatum* strain LS 03 (KJ721990)	99	KP018216
Pa-1-4-3	*Trichoderma aureoviride* strain SL (KJ610807)	99	KP018217
Pa-1-6-1	*Alternaria rosae* strain CM24T-EY-E (KF815569)	99	KP018218
Pa-1-6-2	*Lewia* sp. OUCMBI101191 (HQ914885)	100	KP018219
Pa-1-8-1	*Alternaria alternata* strain HMA1D (KJ677246)	100	KP018220
Pa-1-8-2	*Penicillium oxalicum* strain SY20-5 (KJ619622)	99	KP018221
Pa-1-8-3	*Fusarium incarnatum* strain FI-00602 (KJ572780)	100	KP018222
Pa-1-8-4	*Talaromyces verruculosus* (JN676121)	99	KP018223
Pa-1-9-1	*Fusarium incarnatum* strain FI-00602 (KJ572780)	100	KP018224
Pa-1-9-2	*Alternaria alternata* strain HMA1D (KJ677246)	100	KP018225
Pa-1-9-3	*Alternaria* sp. HT-M18-LS (KJ527010)	100	KP018226
Pa-2-2-2	*Lewia* sp. OUCMBI101191 (HQ914885)	99	KP018227
Pa-2-2-3	*Simplicillium obclavatum* (AB604000)	100	KP018228
Pa-2-2-4	*Macrophoma* sp. TXc4-6 (HQ262514)	100	KP018229
Pa-2-3-1	*Alternaria* sp. DX-FOF7 (KF558883)	100	KP018230
Pa-2-3-4	*Alternaria alternata* strain HMA1D (KJ677246)	100	KP018231
Pa-2-4-1-2	*Penicillium* sp. CCF3828 (FJ430753)	99	KP018232
Pa-2-4-2	*Trichoderma ovalisporum* strain (KC847168)	100	KP018233
Pa-2-5-2	*Penicillium* sp. CCF3828 (FJ430753)	99	KP018234
Pa-2-6-2	*Alternaria alternata* strain HMA1D (KJ677246)	100	KP018235
Pa-2-6-5	*Cladosporium* sp. HT-Z1-V (KJ527013)	100	KP018236
Pa-2-7-1	*Phoma* sp. P17E3 (JN207293)	99	KP018237
Pa-2-7-3	*Alternaria alternata* strain HMA1D (KJ677246)	100	KP018238
Pa-2-8-1-2	*Fusarium caeruleum* (KF887087)	100	KP018239
Pa-2-8-2	*Fusarium oxysporum* strain P43 (JX045812)	100	KP018240
Pa-2-8-3-1	*Fusarium incarnatum* strain LS 03 (KJ721990)	100	KP018241
Pa-2-9-1	*Verticillium saksenae* (KF472156)	99	KP018242
Pa-2-9-2	*Lewia* sp. OUCMBI101191 (HQ914885)	100	KP018243
Pa-2-9-3	*Sclerostagonospora phragmiticola* strain (KF251230)	100	KP018244
Pa-2-9-4	*Cladosporium cladosporioides* strain (KJ589558)	100	KP018245
Pa-2-9-5	*Verticillium saksenae* (KF472156)	99	KP018246
Pa-2-9-7	*Lewia* sp. OUCMBI101191 (HQ914885)	100	KP018247
Pa-2-9-8	*Lewia* sp. OUCMBI101191 (HQ914885)	100	KP018248
Pa-2-10-2	*Fusarium longipes* (HG423537)	99	KP018249
Pa-2-10-6	*Lewia* sp. OUCMBI101191 (HQ914885)	100	KP018250
Pa-3-1-1	*Alternaria alternata* strain HMA1D (KJ677246)	100	KP018251
Pa-3-1-3	*Fusarium longipes* (HG423537)	99	KP018252
Pa-3-2-3	*Fusarium longipes* (HG423537)	99	KP018253
Pa-3-2-4	*Talaromyces verruculosus* (JN676121)	100	KP018254
Pa-3-3-1	*Phoma* sp. P17E3 (JN207293)	99	KP018255
Pa-3-4-1	*Fusarium incarnatum* strain LS 03 (KJ721990)	100	KP018256
Pa-3-6-2	*Fusarium incarnatum* strain LS 03 (KJ721990)	100	KP018257
Pa-3-7-2	*Fusarium commune* strain dH 23113 (JX162390)	100	KP018258
Pa-3-7-3	*Penicillium spinulosum* isolate FFJC 16 (KF876837)	100	KP018259
Pa-3-7-4-1	*Cladosporium cladosporioides* isolate (KJ572146)	100	KP018260
Pa-3-7-4-2	*Phoma* sp. P17E3 (JN207293)	99	KP018261
Pa-3-9-1	*Fusarium commune* strain dH 23113 (JX162390)	100	KP018262
Pa-3-10-1	*Lecanicillium* sp. O_3_BESC_246b (KC007329)	100	KP018263
Pa-3-10-2	*Fusarium longipes* (HG423537)	99	KP018264
Pa-3-10-3	*Lewia* sp. OUCMBI101191 (HQ914885)	100	KP018265
Sa-1-3-1	*Fusarium oxysporum* strain HPA2 (KJ677253)	100	KP018266
Sa-1-3-2	*Aspergillus brasiliensis* strain HPA8 (KJ677257)	100	KP018267
Sa-1-4-3	*Acrostalagmus luteoalbus* isolate AcLu2 (JQ387575)	100	KP018268
Sa-1-5-1	*Phoma* sp. JX1203 (KC203049)	100	KP018269
Sa-1-5-2-2	*Phoma* sp. JX1203 (KC203049)	100	KP018270
Sa-1-6-2	*Macrophoma* sp. TXc4-6 (HQ262514)	100	KP018271
Sa-1-7-2	*Macrophoma* sp. TXc4-6 (HQ262514)	100	KP018272
Sa-1-9-1	*Fusarium incarnatum* strain FI-00602 (KJ572780)	100	KP018273
Sa-1-9-2	*Macrophoma* sp. TXc4-6 (HQ262514)	100	KP018274
Sa-2-1-2	*Alternaria alternata* strain HMA1D (KJ677246)	100	KP018275
Sa-2-2-1	*Exophiala oligosperma* (AB480204)	100	KP018276
Sa-2-2-1-1	*Paraconiothyrium cyclothyrioides* strain (KC215138)	100	KP018277
Sa-2-2-3	*Exophiala oligosperma* (AB777520)	100	KP018278
Sa-2-3-1	*Macrophoma* sp. TXc4-6 (HQ262514)	100	KP018279
Sa-2-3-2	*Macrophoma* sp. TXc4-6 (HQ262514)	100	KP018280
Sa-2-4-1	*Pleospora bjoerlingii* (JX045842)	100	KP018281
Sa-2-6-2	*Penicillium canescens* strain CV0198 (JX140832)	100	KP018282
Sa-2-7-1	*Plectosphaerella* sp. MF-1 (AB520859)	100	KP018283
Sa-2-7-2	*Aspergillus terreus* isolate D34 (KF971363)	100	KP018284
Sa-2-8-1	*Penicillium lapidosum* (KJ676451)	100	KP018285
Sa-2-9-1	*Sclerostagonospora phragmiticola* strain (KF251230)	99	KP018286
Sa-2-9-4	*Paraphoma* sp. BJ18 (KJ702586)	100	KP018287
Sa-2-10-1	*Macrophoma* sp. TXc4-6 (HQ262514)	100	KP018288
Sa-2-10-2	*Alternaria* sp. BJ35 (KJ702610)	100	KP018289
Sa-3-1-1	*Hypocrea* sp. SFCF20120803-50 (KF313111)	100	KP018290
Sa-3-1-2	*Aspergillus* aff. *fumigatus* A28 (JN246065)	100	KP018291
Sa-3-2-2	*Cladosporium* sp. XJ18 (KF143793)	100	KP018292
Sa-3-3-1	*Acrostalagmus luteoalbus* strain PTV-1 (GU813970)	100	KP018293
Sa-3-4-2	*Macrophoma* sp. TXc4-6 (HQ262514)	100	KP018294
Sa-3-4-3	*Macrophoma* sp. TXc4-6 (HQ262514)	100	KP018295
Sa-3-6-1	*Penicillium citrinum* strain NF7 (KJ653821)	100	KP018296
Sa-3-6-3	*Fusarium oxysporum* isolate F102 (KJ512160)	100	KP018297
Sa-3-8-1	*Penicillium simplicissimum* strain (KF815055)	99	KP018298
Sa-3-8-1-1	*Alternaria* sp. BJ35 (KJ702610)	100	KP018299
Sa-3-8-2-1	*Colletotrichum gloeosporioides* strain (KJ632430)	100	KP018300
Sa-3-8-3	*Aspergillus* sp. BJ39 (KJ702608)	100	KP018301
Sa-3-9-1	*Fusarium oxysporum* isolate F102 (KJ512160)	100	KP018302
Sa-3-9-2	*Fusarium oxysporum* strain HPA2 (KJ677253)	99	KP018303
Sa-3-10-1	*Penicillium citrinum* strain NF7 (KJ653821)	100	KP018304
Sa-3-10-2	*Trichoderma harzianum* (HG940484)	100	KP018305
Lt-1-1-1	*Pestalotiopsis* sp. 1 AE-2013 strain F4872 (KF746123)	100	KP018306
Lt-1-2-2	*Pleospora bjoerlingii* (JX045842)	100	KP018307
Lt-1-3-2	*Penicillium lapidosum* (KJ676451)	100	KP018308
Lt-1-4-1	*Fusarium longipes* (KJ412506)	100	KP018309
Lt-1-5-1	*Paraconiothyrium cyclothyrioides* strain (KC215138)	100	KP018310
Lt-1-6-1	*Pleospora bjoerlingii* (JX045842)	100	KP018311
Lt-1-8-2	*Alternaria alternata* strain SR/I/90 (KJ767532)	100	KP018312
Lt-1-9-1	*Fusarium oxysporum* f. sp. conglutinans (KF381081)	100	KP018313
Lt-1-10-1	*Pestalotiopsis clavispora* strain P44 (JX045813)	100	KP018314
Lt-1-10-2	*Cochliobolus kusanoi* isolate SH8 (KJ572135)	100	KP018315
Lt-2-1-1	*Colletotrichum acutatum* strain 11E031 (KF717039)	100	KP018316
Lt-2-4-1	*Penicillium paneum* strain M-18 (JQ422610)	100	KP018317
Lt-2-5-1-1	*Phoma* sp. EIODSF018 (KJ173541)	100	KP018318
Lt-2-5-1-2	*Trichoderma* sp. BCC 3579 (AY550911)	99	KP018319
Lt-2-6-2	*Penicillium* sp. SK14JW2P (KC545799)	100	KP018320
Lt-2-7-1	*Alternaria* sp. BJ35 (KJ702610)	100	KP018321
Lt-2-8-2	*Macrophoma* sp. TXc4-6 (HQ262514)	100	KP018322
Lt-3-2-2	*Trichoderma harzianum* strain ML16-1 (KJ619615)	100	KP018323
Lt-3-3-1	*Lewia* sp. OUCMBI101191 (HQ914885)	100	KP018324
Lt-3-3-2	*Meira* sp. JCM 18504 (AB778892)	99	KP018325
Lt-3-6-1	*Penicillium* sp. 12140 (JX657339)	100	KP018326
Lt-3-7-1	*Alternaria* sp. BJ35 (KJ702610)	100	KP018327
Lt-3-9-1	*Alternaria* sp. BJ35 (KJ702610)	99	KP018328
Lt-3-9-2	*Aspergillus brasiliensis* strain HPA8 (KJ677257)	100	KP018329
Lt-3-9-4	*Aspergillus clavatus* strain USMO08 (KF669482)	99	KP018330
Su-1-1-1	*Talaromyces pinophilus* isolate SCLB5 (KF913534)	100	KP018331
Su-1-2-1	*Talaromyces pinophilus* isolate SCLB5 (KF913534)	100	KP018332
Su-1-2-2	*Penicillium* sp. OY18307 (FJ571475)	100	KP018333
Su-1-4-1	*Hypocrea* sp. SFCF20120803-50 (KF313111)	100	KP018334
Su-1-6-2	*Fusarium* sp. BJ9 (KJ702598)	100	KP018335
Su-1-6-2-1	*Alternaria* sp. DX-FOF7 (KF558883)	100	KP018336
Su-1-7-2	*Phomopsis* sp. H4243 (GU595056)	99	KP018337
Su-1-10-2	*Alternaria* sp. BJ35 (KJ702610)	100	KP018338
Su-2-1-1	*Pleospora bjoerlingii* (JX045842)	100	KP018339
Su-2-2-3	*Purpureocillium lilacinum* strain (KC157754)	100	KP018340
Su-2-3-1	*Phoma* sp. Y19 (KJ572232)	100	KP018341
Su-2-3-2	*Pleospora bjoerlingii* (JX045842)	100	KP018342
Su-2-4-3	*Purpureocillium lilacinum* strain (KC157756)	100	KP018343
Su-2-5-3	*Aspergillus brasiliensis* (KJ445022)	100	KP018344
Su-2-6-2	*Pleospora bjoerlingii* (JX045842)	100	KP018345
Su-2-7-1	*Gibberella fujikuroi* (KC752592)	100	KP018346
Su-2-7-2	*Penicillium* sp. OY18307 (FJ571475)	100	KP018347
Su-2-8-1	*Alternaria* sp. BJ35 (KJ702610)	100	KP018348
Su-2-8-2	*Metacordyceps chlamydosporia* (FN598950)	99	KP018349
Su-2-9-1	*Penicillium* sp. Cs/13/2 (JN585948)	100	KP018350
Su-2-9-2	*Alternaria* sp. BJ35 (KJ702610)	100	KP018351
Su-2-9-3	*Macrophoma* sp. TXc4-6 (HQ262514)	100	KP018352
Su-2-10-1	*Alternaria* sp. 174wat (KF811432)	100	KP018353
Su-2-10-2	*Penicillium* sp. KJ-2012 strain GZU(JQ965022)	100	KP018354
Su-3-1-1	*Davidiella macrospora* (KJ529009)	100	KP018355
Su-3-1-1-1	*Davidiella macrospora* (KJ529009)	100	KP018356
Su-3-1-2	*Pleospora bjoerlingii* (JX045842)	99	KP018357
Su-3-3-1	*Fusarium oxysporum* strain P43 (JX045812)	100	KP018358
Su-3-4-1	*Pestalotiopsis clavispora* strain P44 (JX045813)	100	KP018359
Su-3-4-3	*Pleospora bjoerlingii* (JX045842)	100	KP018360
Su-3-5-1	*Phomopsis* sp. H4243 (GU595056)	99	KP018361
Su-3-5-2	*Aspergillus terreus* isolate D34 (KF971363)	100	KP018362
Su-3-6-1	*Colletotrichum gloeosporioides* strain (KJ632430)	100	KP018363
Su-3-6-3	*Aspergillus allahabadii* strain CBS (GQ342626)	100	KP018364
Su-3-6-5	*Pestalotiopsis clavispora* strain P44 (JX045813)	100	KP018365
Su-3-7-2	*Purpureocillium lilacinum* strain E303 (KJ540087)	100	KP018366
Su-3-7-3	*Cladosporium* sp. BJ45 (KJ702611)	100	KP018367
Su-3-9-1	*Clonostachys rosea* strain F-3-51 (KF887020)	100	KP018368
Su-3-9-2	*Gliomastix murorum* (AB540558)	100	KP018369
Su-3-10-1	*Alternaria* sp. BJ35 (KJ702610)	100	KP018370
Su-3-10-2-1	*Colletotrichum gloeosporioides* strain (KJ632430)	100	KP018371
Sm-1-1-2	*Aspergillus* sp. SL-F20 (KJ528990)	99	KP018372
Sm-1-3-1	*Macrophoma* sp. TXc4-6 (HQ262514)	100	KP018373
Sm-1-3-2	*Tetracladium setigerum* isolate (HQ647302)	99	KP018374
Sm-1-5-3	*Macrophoma* sp. TXc4-6 (HQ262514)	100	KP018375
Sm-1-6-2	*Alternaria* sp. BJ35 (KJ702610)	100	KP018376
Sm-1-9-2-2	*Macrophoma* sp. TXc4-6 (HQ262514)	100	KP018377
Sm-1-10-1	*Fusarium andiyazi* strain CBS 134430 (KC954400)	100	KP018378
Sm-1-10-2	*Alternaria* sp. BJ35 (KJ702610)	100	KP018379
Sm-2-1-3	*Alternaria* sp. BJ35 (KJ702610)	100	KP018380
Sm-2-6-1	*Macrophoma* sp. TXc4-6 (HQ262514)	100	KP018381
Sm-2-6-2	*Alternaria* sp. BJ35 (KJ702610)	100	KP018382
Sm-2-7-2	*Macrophoma* sp. TXc4-6 (HQ262514)	100	KP018383
Sm-2-8-1	*Talaromyces trachyspermus* strain (KF147920)	99	KP018384
Sm-2-8-2-1	*Exophiala oligosperma* (AB777520)	100	KP018385
Sm-2-8-2-2	*Meira* sp. JCM 18504 (AB778892)	99	KP018386
Sm-2-9-1	*Alternaria* sp. DX-FOF7 (KF558883)	100	KP018387
Sm-2-9-2	*Alternaria* sp. BJ35 (KJ702610)	100	KP018388
Sm-2-10-1	*Alternaria* sp. HT-M18-LS (KJ527010)	100	KP018389
Sm-2-10-2	*Cladosporium cladosporioides* strain (KJ589558)	100	KP018390
Sm-3-1-1	*Macrophoma* sp. TXc4-6 (HQ262514)	99	KP018391
Sm-3-1-3	*Cladosporium oxysporum* strain B2F2 (KJ589590)	100	KP018392
Sm-3-2-1	*Alternaria* sp. BJ35 (KJ702610)	100	KP018393
Sm-3-3-3	*Penicillium* sp. JMG302 (KJ598874)	98	KP018394
Sm-3-4-1	*Cochliobolus kusanoi* isolate SH8 (KJ572135)	100	KP018395
Sm-3-5-1	*Alternaria* sp. DX-FOF7 (KF558883)	100	KP018396
Sm-3-8-1	*Alternaria* sp. DX-FOF7 (KF558883)	100	KP018397
Sm-3-8-2	*Macrophoma* sp. TXc4-6 (HQ262514)	100	KP018398
Sm-3-9-2	*Macrophoma* sp. TXc4-6 (HQ262514)	100	KP018399
Sm-3-10-2	*Alternaria* sp. HT-M18-LS (KJ527010)	100	KP018400
Sm-3-10-3	*Cladosporium cladosporioides* strain (KJ589558)	100	KP018401

**Table 3:** Endophytic fungi (188 strains) isolated from five plants with scientific names, plant codes, taxa of fungal strain, and number of fungal isolates.

Scientific name of plant sample	Abbreviated plant name	Taxon of fungal strains	No. of isolates
*Phragmites australis*	Pa	14 genera, 16 species	52
*Suaeda australis*	Sa	17 genera, 17 species	40
*Limonium tetragonum*	Lt	14 genera, 13 species	25
*Suaeda glauca* Bunge	Su	19 genera, 14 species	41
*Suaeda maritima*	Sm	11 genera, 7 species	30

Pa, *Phragmites australis*; Sa, *Suaeda australis*; Lt, *Limonium tetragonum*; Su, *Suaeda glauca* Bunge; and Sm, *Suaeda maritima*.

**Table 4:** Diversity indices and distribution of endophytic fungi isolated from plants native to the Buan salt marsh.

Fungal taxon	Pa	Sa	Lt	Su	Sm
*Acrostalagmus*		2			
*Alternaria*	10	3	4	6	11
*Aspergillus*		4	2	3	1
*Cladosporium*	3	1		1	3
*Clonostachys*				1	
*Cochliobolus*			1		1
*Colletotrichum*		1	1	2	
*Davidiella*				2	
*Exophiala*		2			1
*Fusarium*	14	5	2	2	1
*Gibberella*				1	
*Gliomastix*				1	
*Hypocrea*		1		1	
*Lecanicillium*	1				
*Lewia*	7		1		
*Macrophoma*	1	8	1	1	8
*Meira*			1		1
*Metacordyceps*				1	
*Paraconiothyrium*		1	1		
*Paraphoma*		1			
*Penicillium*	4	5	4	4	1
*Pestalotiopsis*	1		2	2	
*Phoma*	3	2	1	1	
*Phomopsis*				2	
*Plectosphaerella*		1			
*Pleospora*		1	2	5	
*Purpureocillium*				3	
*Sclerostagonospora*	1	1			
*Simplicillium*	1				
*Talaromyces*	2			2	1
*Tetracladium*					1
*Trichoderma*	2	1	2		
*Verticillium*	2				
N	52	40	25	41	30
S	14	17	14	19	11
Shannon diversity index (*H'*)	2.22	2.55	2.50	2.76	1.86
Simpson’s index of diversity (*1-D*)	0.904	0.936	0.967	0.970	0.929
Menhinick's index (*Dmn*)	1.94	2.69	2.80	2.97	2.01
Margalef's index (*Dmg*)	3.29	4.34	4.04	4.85	2.94
Fisher's diversity (*α*)	6.29	11.17	13.14	13.75	6.26

Pa, *Phragmites australis*; Sa, *Suaeda australis*; Lt, *Limonium tetragonum*; Su, *Suaeda glauca* Bunge; and Sm, *Suaeda maritima*.
